# Supplementary material for: The impact of skeletal muscle disuse on distinct echo intensity bands: A retrospective analysis
Source: PLoS One. 2022 Jan 11;17(1):e0262553. doi: 10.1371/journal.pone.0262553 (PMC8752001; doi:10.1371/journal.pone.0262553)
Supplement: Attachment — Submitted filename: Response to reviewers #1.docx [file pone.0262553.s002.docx]

**Responses to Comments from Reviewers – Article ID: PONE-D-21-29548**

*We would like to thank the reviewers and editorial staff at PLOS One for taking the time to review our work and provide feedback. The comments that were provided were thoughtful and will improve this manuscript. Our responses to the reviewers’ comments are below in italicized font. Changes to the manuscript are highlighted yellow in the Revised Manuscript with Track Changes document.*

**Reviewer #1:**

*Thank you for taking the time to review our manuscript. Your comments are well received and have added valuable improvements to this article. Please find our replies below and let us know if you have any more input.*

L18. I don’t think this sentence is completely correct. EI is measured from ultrasound. I suggest stating something like “… EI can be assessed quickly from ultrasound scans”.

- *Thank you for pointing out this important distinction! We have edited the manuscript to include this exact wording. It now says, “In addition to measures of muscle size, EI can be assessed quickly from ultrasound scans (14).”*

L18-22. This is true if ImageJ is used as a software for the analysis. This should be acknowledged.

- *Of course! We are glad you mentioned this. To make this more clear we simply added “Using ImageJ software,” to this sentence. It now says, “Using ImageJ software, EI exists on a scale of 0-255 arbitrary units (A.U.) in which skeletal muscle tissue appears black (“hypoechoic”, EI=0) and non-contractile tissues (i.e., intramuscular adipose and fibrous tissue) appears white (“hyperechoic”, EI=255).”*
- *We hope this change helps and if there is anything else we can change, we will do it.*

L103-106. Please, provide further details about the technique used for EFOV images acquisition.

- *Thank you for this comment. We agree this will be helpful for readers, especially those seeking to replicate the methods. This section now says, “In accordance with previous studies, images were captured at 50% of the length between the greater trochanter and the lateral femoral condyle (18).* *During femur measurement, markings were placed on the skin at the level to be imaged. A dense foam pad was strapped onto the leg at the site to guide the ultrasound probe. Water-soluble transmission gel (Aquasonic 100 ultrasonography transmission gel, Parker Laboratories, Inc., Fairfield, NJ, USA) was applied to the skin* *along the probe path to enhance transducer coupling during imaging.”*
- *This type of change to the manuscript will certainly help others understand and replicate our methods.*

L189. At this point of the manuscript there is no need to state that a difference is “significant”. I suggest erasing it.

- *You are right. We have erased “significant” from this line. It now reads, “Analysis of the 50 A.U. change scores indicated that these differences were large.”*

L201. I am not a native English speaker, so consider this suggestion only if it is correct: “… the work by Pinto and Pinto..”

- *This suggestion makes sense! We have added the exact wording you suggested. It now says, “To our knowledge, the work by Pinto and Pinto (18) was the first to question whether more information can be obtained by scrutinizing separate regions of the traditional 0-255 A.U. histogram.”*

L209. Can you make a speculation about it?

- *Internally we have speculated, but want to be careful about confusing readers. We have cautiously added to this section in order to offer a possible explanation while also not making definitive claims. This section now says, “The reason(s) for the large change in the % EI_101-150_ band, but not the %EI_51-100_ band, is unclear. We speculate that, as fat and/or fibrous tissue entered the muscle, pixels in each band shifted to higher bands. For example, a large number of pixels may have left the %EI_0-50_ band and entered the %EI_51-100_ band. If there is a cascading effect, it is possible that enough pixels left the %EI_51-100_ band to increase the number in the %EI_101-150_ band, but those leaving the %EI_51-100_ band were replaced by pixels from the %EI_0-50_ band. This seems logical; however, it is not known whether the explanation is so simple and it requires further study.”*
- *We hope this speculation is sufficient. Please let us know if you have more thoughts or suggestions.*

L229. Same as at L201.

- *Thank you for this suggestion. We have changed these sentences in a similar way.*

L245. Same as at L201.

- *Thank you for this suggestion. We have changed these sentences in a similar way.*

**Reviewer #2:**

*Thank you for your time and effort in reviewing our manuscript! We are happy to work together. Your thoughts and suggestions are of great value and have significantly improved this paper. Please find our comments below in italics.*

The present manuscript aimed to investigate changes in muscle quality after two weeks of lower limb immobilization comparing the traditional EI mean with EI bands approach. The paper is well written and the methodological research question is of interest. I only have some minor concerns that need to be addressed:

Abstract

I think your abstract conclusion should highlight also the comparison between 25 and 50 band intervals.

- *This is a great suggestion! We have added a short sentence that describes the comparison. This section now says, “**Further analysis of the 25 A.U. bands indicated that the percentage of pixels within the 25-50 A.U. band decreased (-2.97 ± 3.64%), whereas the 101-125 (1.62 ± 1.47%) and 126-150 A.U. (1.18 ± 1.07%) bands increased. Comparison of the 50 A.U. and 25 A.U. band methods found that 25 A.U. bands offer little additional insight. Though studies are needed to ascertain the factors that may influence specific bands, changes in EI during muscle disuse are not homogeneous across the pixel histogram.”*
- *We hope this addition makes the conclusion more clear and please let us know if you have further suggestions.*

In the last sentence of the abstract, please specify that you are referring specifically to EI mean, otherwise this sentence could be misinterpreted (as EI is not robust in general)

- *Thank you for this comment. We have added to this sentence to make it clear we are referring to EImean. This sentence now reads, “We encourage investigators to think critically about the robustness of data obtained from EI histograms, rather than simply reporting the EI_mean_ value, in muscle quality research.”*

L46-48: have you tried to carry out the correlation between VL CSA changes and EI in the different bands?

- *This is a very good question. We have not done the correlation. This is a great idea for future research; however, in our internal discussion we decided this would be outside the scope of the paper.*

L209: Could you add at least some speculation on the potential physiological reasons?

- *This is a difficult but important comment! We want to be careful, but believe we can speculate about the physiological reasons in the context of the measurement. We have added to this section, “The reason(s) for the large change in the % EI_101-150_ band, but not the %EI_51-100_ band, is unclear. We speculate that, as fat and/or fibrous tissue entered the muscle, pixels in each band shifted to higher bands. For example, a large number of pixels may have left the %EI_0-50_ band and entered the %EI_51-100_ band. If there is a cascading effect, it is possible that enough pixels left the %EI_51-100_ band to increase the number in the %EI_101-150_ band, but those leaving the %EI_51-100_ band were replaced by pixels from the %EI_0-50_ band. This seems logical; however, it is not known whether the explanation is so simple and it requires further study.*
- *We hope this addition is sufficient. Please let us know if you have further thoughts or suggestions.*

L233: Why did you limit your analysis to VL? From Figure 2 the rectus femoris (RF) seems to be fully visible. I think that a comparison between VL and RF would be a nice addition to this work, considering that RF (being multi-joint) is generally less affected by disuse compared to the VL.

- *Thank you for this comment. This is an important question that we discussed extensively within our group, and we decided not to include RF. The primary purpose of this retrospective analysis was to explore the EI band technique. Because of this, we feel it was most appropriate to limit our application of the technique to a muscle that we hypothesized would be most likely to change. Further, in our disuse study we only immobilized the knee joint but the RF is multi-joint. When participants walked on crutches, they certainly used their RF for hip flexion to swing the suspended limb- helping with forward propulsion. We believe this repetitive RF activity would confound any changes found in EI with further analysis.*
